# Chondroitin Sulfate Nanovectorized by LC-PUFAs Nanocarriers Extracted from Salmon (*Salmo salar*) by Green Process with Decreased Inflammatory Marker Expression in Interleukin-1β-Stimulated Primary Human Chondrocytes In Vitro Culture

**DOI:** 10.3390/md22120571

**Published:** 2024-12-20

**Authors:** Louis Pruvost, Maureen Gerlei, Cédric Paris, Émilie Velot, Cyril J.-F. Kahn, Arnaud Bianchi, Michel Linder

**Affiliations:** 1 LIBio, Université de Lorraine, F-54000 Nancy, France; louis.pruvost@univ-lorraine.fr (L.P.); maureen.gerlei@univ-lorraine.fr (M.G.); cedric.paris@univ-lorraine.fr (C.P.); cyril.kahn@univ-lorraine.fr (C.J.-F.K.); 2 CNRS, IMoPA, Université de Lorraine, F-54000 Nancy, France; emilie.velot@univ-lorraine.fr (É.V.); arnaud.bianchi@univ-lorraine.fr (A.B.)

**Keywords:** liposome, chondroitin sulfate, phospholipid, *Salmo salar*, enzymatic hydrolysis, anti-inflammatory, encapsulation

## Abstract

Chondroitin sulfate (CS), a glycosaminoglycan, supports health through various physiological functions, including tissue protection, bone growth, and skin aging prevention. It also contributes to anticoagulant or anti-inflammatory processes, with its primary clinical use being osteoarthritis treatment. This study presents the results of the valorization of lipids and CS, both extracted from salmon co-products through enzymatic processes. The polar lipids, naturally rich in long-chain fatty acids (docosahexaenoic acid DHA C22:6 n-3 and eicosapentaenoic acid EPA C20:5 n-3), and the CS, primarily located in the nasal cartilage, were separated and concentrated before being characterized using various techniques to determine functional and lipid composition. These compounds were then used to formulate liposomes of 63 to 95 nm in size composed of 19.38% of DHA and 7.44% of EPA and encapsulating CS extract with a Δdi-4S/Δdi-6S ratio of 0.53 at 2 weight masses (10–30 kDa and >30 kDa) or CS standard all at two different concentrations. Liposomes were tested on human chondrocytes in inflamed conditions. Thus, compatibility tests, the expression of various inflammation markers at transcriptional and molecular levels, nitrites, and the amount of collagenase produced were analyzed. The results showed that CS, in synergy with the liposomes, played a positive role in combating chondrocyte inflammation even at a low concentration.

## 1. Introduction

According to the Food and Agriculture Organization of the United Nations (FAO) report “The State of World Fisheries and Aquaculture” (2024) [1], the fisheries and aquaculture sector (mainly the production of fish, mollusks, and crustaceans) accounted for 185 million tons globally in 2022. This production generated 20 million tons of by-products (heads, viscera, skeletons) [2], which represent a very interesting and widely available source for biomolecule extraction. This is particularly true for salmon, which is highly consumed worldwide [1]. The heads generated by the aquaculture sector (*Salmo salar*) are a source of long-chain polyunsaturated fatty acids (LC-PUFAs), mainly eicosapentaenoic acid (EPA; C20:5 n-3) and docosahexaenoic acid (DHA; C22:6 n-3), as well as cartilage rich in chondroitin sulfate (CS). These LC-PUFAs, present in the form of triacylglycerols but also polar lipids (phospholipids), have numerous preventive and therapeutic effects on brain function, cardiovascular diseases, and inflammation. Current recommendations for EPA and DHA intake for general health are typically 400 to 500 mg/day as a combination of both fatty acids (French Agency of Food Administration, AFSSA) [3].

Moreover, salmon heads are rich in polar lipids, particularly phospholipids, which are highly valued for their ability to form liposomes due to their amphipathic properties [4]. In most cases, the core of the vesicle and its surface are hydrophilic, thanks to lipids that self-assemble to form a lipid bilayer, while the space between the two layers of phospholipids is hydrophobic. However, some liposomes can be unilamellar [5]. This particular structure allows for the encapsulation of hydrophilic molecules in the aqueous core or hydrophobic molecules in the membrane. These molecules can contain therapeutic or nutritional active ingredients for applications in cosmetics, pharmacology, or even for the food industry [6].

Glycosaminoglycans from salmon cartilage are increasingly being studied for their interesting properties in attenuating inflammatory responses, limiting angiogenesis, and aging phenomena [7]. Found in the cartilage of the salmon’s nasal area, glycosaminoglycans are negatively charged polysaccharides, classified based on the sugars that make up their structure [8].

Among these compounds, CS has a wide range of physiological functions and clinical applications [9]. CS exhibits anticoagulant [10], antioxidant [11], antibacterial [12], and anti-inflammatory activities [13]. The most common clinical application of CS is in the treatment of osteoarthritis [14]. Osteoarthritis is the most common musculoskeletal disease, associated with cartilage degeneration. More than 300 million patients worldwide are affected [15]. CS is part of the SYSADOA (Symptomatic Slow-Acting Drugs for Osteoarthritis) treatments [16]. Indeed, even though the osteoarthritis mechanisms are not fully understood, it was reported in the literature that low-grade joint inflammation participates in this progressive disease [17,18]. Pro-inflammatory cytokines, such as interleukin (IL)-1β, promote the degeneration of the hyaline extracellular matrix by increasing its degradation and preventing its synthesis. Thus, this catabolic process induces an articular chondrocyte altered phenotype and prevents cells from restoring the extracellular matrix equilibrium [10]. Cyclooxygenase (COX)-2, microsomal prostaglandin E2 synthase (mPGES-1), and inducible nitric oxide synthase (iNOS) are among the mediators of inflammation induced by IL-1β [19,20]; monitoring their level of expression reflects the level of inflammation induced by IL-1β. Moreover, COX-2 and mPGES-1 are both responsible for prostaglandin E2 (PGE2) synthesis, while iNOS produces nitric oxide (NO), which both take part in extracellular matrix catabolism, leading to cartilage degradation [21,22]. In addition, matrix metalloproteinases (MMPs), which are responsible for the degenerative part in osteoarthritis pathology, are also good markers of degeneration activity after inflammatory stimulation.

CS is composed of repeating disaccharide units [→4)-β-D-GlcA-(1→3)-β-D-GalNAc-(1→]_n_, where GlcA corresponds to glucuronic acid and GalNAc to N-acetylgalactosamine (Table 1). The disaccharide units can be classified into the following five groups based on the arrangement of sulfate groups within the disaccharide (number and position of sulfate groups): GlcA-GalNAc (CS-0 unit), GlcA-GalNAc(4S) (CS-A unit), GlcA-GalNAc(6S) (CS-C unit), GlcA(2S)-GalNAc(6S) (CS-D unit), and GlcA-GalNAc(4S,6S) (CS-E unit) [7].

The degree of sulfation and the molecular weight of CS chains can vary depending on the tissue of origin, the age, and the species of the animal. Generally, tissues from terrestrial animals are richer in CS-0 and CS-A units (>60%) and have lighter CS chains (14 to 26 kDa). For marine species, CS chains are heavier (up to 70 kDa), with more complex disaccharides like CS-C, CS-D, and CS-E (proportions vary depending on the species) [23].

**Table 1:** Specific information regarding CS bioactivities.

Molecule	Formula	Sources	Potential Application	References
CS-O	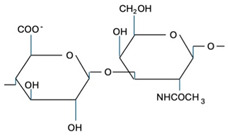	Shark fins, human, synthetic	Anti-inflammatory, osteoarthritis, biotechnology precursor	[24,25,26]
CS-A	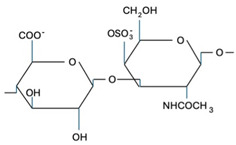	Shark fins, bovine trachea, sturgeon notochord	Anti-inflammatory, repair the central nervous system, malarial vaccine	[27,28,29]
CS-C	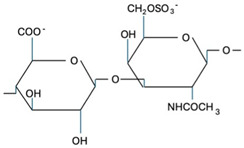	Unknown	Anti-tumor	[30]
CS-E	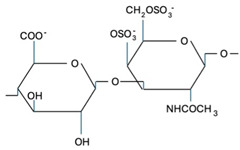	Unknown	Anti-thrombus, anti-viral, anti-inflammatory	[31,32,33]

Since liposomes have increased the bioavailability of molecules (transforming growth factor beta 1 (TGF-β1), antioxidants), chondrocyte in particular [34,35,36], the choice was made to vectorize CS to decrease the inflammatory effect of IL-1β. In addition, the properties of nanoliposomes were enhanced by the choice of phospholipid origin. Indeed, marine phospholipids are naturally rich in LC-PUFAs such as DHA, also known to have a reduced inflammation effect [37,38,39].

The objective of the present study is to perform an enzymatic extraction of chondroitin sulfate (CS) and polar lipids from salmon heads, characterize them, and then formulate liposomes naturally rich in LC-PUFAs. Fractions of CS obtained through ultrafiltration will be encapsulated within these liposomes. These liposome formulations are then applied to chondrocytes cultured under inflammatory conditions to study their ability to decrease the expression of inflammatory markers.

## 2. Results

### 2.1. FT-IR Spectroscopy of CS and Lipids Analyses

**Figure 1:**
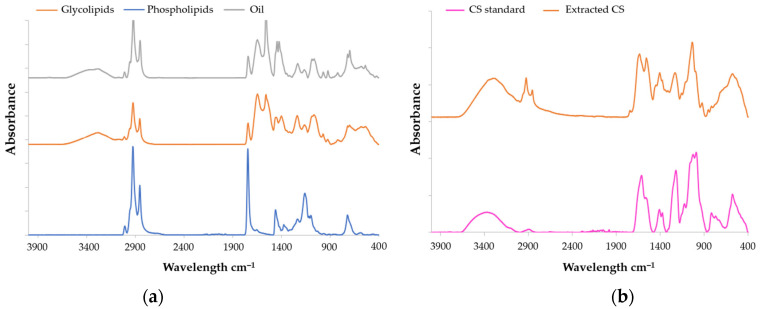
FT-IR spectra of (**a**) CS and (**b**) lipids all extracted from *Salmo salar* heads by enzymatic hydrolysis.

According to Kai-Ruei Yang et al. [40], the Fourier Transform Infrared (FTIR) technique is regularly used to characterize the functional groups of CS. The FTIR spectra of the marine CS standard (Sigma-Aldrich, International standard, Saint-Quentin-Fallavier, France), as well as CS extracted from salmon, were measured from 4000 cm^−1^ to 400 cm^−1^ at a resolution of 6 cm^−1^ (Figure 1b, Table 2). The CS extracted from salmon heads (*Salmo salar*) exhibit a similar spectrum to that of the standard. The peak observed at 3412 cm^−1^ originated from the stretching vibrations of hydroxyl and carboxyl groups, while bands observed at 2920 cm^−1^ and 2850 cm^−1^ were assigned to the stretching and bending vibrations of C-H bonds [41]. The peaks detected at 1410 cm^−1^ and 1625 cm^−1^ were indicative of stretching vibrations associated with C-O bonds, [41]. The bands at, respectively, 1550, 1373, and 1220 cm^−1^ correspond to N-H bending vibration, C-O symmetric vibration, and S-O stretching vibration [42]. According to Garnjanagoonchorn et al. [43], analysis of the spectra of chondroitin-4-sulfate and chondroitin-6-sulfate standards indicated the existence of peaks observed at 857 and 826 cm^−1^. It probably corresponds to the peaks observed at 858 and 820 cm^−1^.

The lipid analyses of salmon by FTIR were conducted using the same parameters as the CS analysis. Lipids spectra are presented in Figure 1a and Table 2. The infrared spectra of the samples exhibited a large absorption band at 3250 cm^−1^ (indicative of hydroperoxides), 3017 cm^−1^ (corresponding to =C−H (*cis*) stretching), 2920 cm^−1^ (attributed to –CH_2_ stretching asymmetry), and 2850 cm^−1^ (associated with methyl –CH_3_ stretching symmetry) according to Wan et al. [44]. Peaks at wavenumber of 1450, 1080, and 720 cm^−1^ correspond, respectively, to CH_2_, CH_3_, esters of the C-O group, and *cis*-disubstituted olefins [44]. 

### 2.2. HPLC-MS Characterization of CS Disaccharides

**Figure 2:**
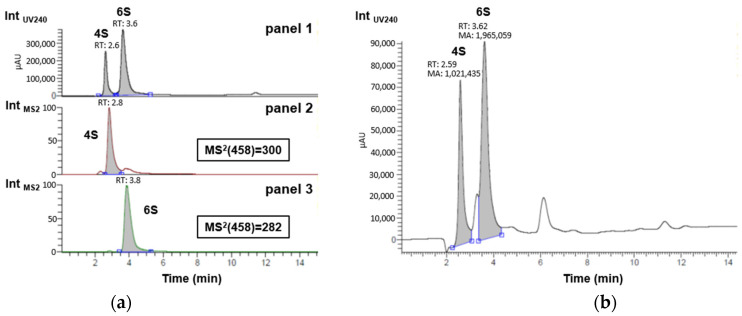
Monitoring Δdi-4S and Δdi-6S disaccharides in (**a**) reference mixture of hydrolyzed commercial marine CS by UV_240_ in panel 1 and by MS^2^ in panels 2 (specific screening of delta Δdi-4S with daughter ion *m*/*z* = 300) and 3 (specific screening of delta Δdi-6S with daughter ion *m*/*z* = 282); and in (**b**) sample of interest by UV_240_ for semi-quantitative evaluation.

A liquid chromatography analysis coupled to UV-Vis absorbance and tandem mass spectrometry (MS/MS) was initially performed on a reference mixture of hydrolyzed CS to unambiguously identify the retention times (RTs) of the two compounds of interest, i.e., Δdi-4S (RTUV_240_ = 2.6 min) and Δdi-6S (RTUV_240_ = 3.6 min) [45]. Then, a robust semi-quantitative UV_240_ study of Δdi-4S and Δdi-6S was performed on our sample assuming a strictly similar response of the two compounds under these conditions and checking beforehand that the peak areas were indeed located in a linear portion of the calibration range (Figure 2b, Appendix A).

As shown in Figure 2b, taking into account only the Δdi-4S and Δdi-6S peaks, their relative percentages are 35% and 65%, respectively. By approximation, taking care of the entire area (with both non-identified peak areas, 222,289 for the peaks between Δdi-4S and Δdi-6S peaks and 345,913 for the peak after Δdi-6S peak) of the sample and not only Δdi-4S and Δdi-6S peaks, it is possible to say that by semi-quantification the extracted sample contains 29% of Δdi-4S and 54% of Δdi-6S approximately. When comparing these results with those presented by Uchisawa et al. [46], the findings are close (30% for Δdi-4S and 60% for Δdi-6S). The composition of disaccharides could likely vary with diet, the origin of the salmon, or the extraction process. It is increasingly recognized that an increase in the proportion of the 6S isomer is linked to enhanced therapeutic efficacy against osteoarthritis. This action is partly based on its anti-inflammatory capacity [33]. Since marine CS samples are rich in the 6S isomer, this explains why they are predominantly used for this application [47]. In the sample, this higher level of Δdi-6S compared to Δdi-4S, typical of marine sources, is observed. The concentration ratio of Δdi-4S/Δdi-6S is 0.53 in the CS extracted from salmon. In the work of Uchisawa et al. [46], the ratio between Δdi-4S and Δdi-6Sis is about 0.51, which is pretty close to the experimental results. These two most common disaccharide categories are increasingly used to link CS activity to their composition [33]. This Δdi-4S/Δdi-6S ratio can therefore be connected to anti-inflammatory activity, as shown by the results in Figures 4 and 5.

### 2.3. Lipid TLC-FID (Iatroscan^®^) Analysis

Phospholipids from *Salmo salar* heads were determined using Iatroscan^®^ in the polar lipid fraction. The results reveal that the fraction contains 14.52 ± 1.41% phosphatidylethanolamine, 1.13 ± 0.11% sterols, 64.66 ± 0.04% phosphatidylcholine, 1.32 ± 0.08% sphingomyelin, 4.77 ± 0.75% lysophosphatidylcholine, and 13.59 ± 2.30% of other polar lipids and non-lipidic compounds.

### 2.4. GC-FID Analysis of Lipids

The fatty acid composition of the three lipid fractions (Triacylglycerol (TAG), glycolipids, and phospholipids) from salmon heads (*Salmo salar*) was determined by gas chromatography coupled with a flame ionization detector (GC-FID) (Table 3) after separation on a silica column. The three fractions exhibit significant differences in their fatty acid composition. The oil fraction contains a high amount of monounsaturated fatty acids (44.10%), particularly oleic acid (28.88%). It also has the highest levels of dienes (8.27%) and trienes (12.01%). However, this fraction is low in long-chain fatty acids such as tetraenes (1.55%) and pentaenes (7.15%). The highest content of saturated fatty acids, mainly composed of palmitic acid in all three fractions, is found in the glycolipids (30.02%). The compositions of phospholipids and glycolipids are very similar and show interesting levels of long-chain fatty acids. The phospholipid fraction has the highest pentaene content (10.59%), primarily composed of EPA (7.44%). It is also the fraction with a significantly higher DHA content compared to the other fractions, with 19.38%. This high level of long-chain fatty acids enhances membrane fluidity in liposomes, facilitating membrane fusion with cells [48].

In comparison, the flesh of Norwegian salmon does not exhibit the same proportions of LC-PUFAs. The DHA and EPA levels are very low (3.94% and 2.19%, respectively). The major fatty acid is oleic acid, at 44%, which is very high. This highlights the advantage of using salmon heads for lipid extraction, as this part of the fish is richer in LC-PUFAs n-3 than the muscle [49]. The high oleic acid content found in the fatty acid composition of salmon (28.88% in oil, 21.02% in glycolipids, and 18.1% in phospholipids) corresponds to the increased intake of vegetable fatty acids in farmed salmon diets. This phenomenon also reduces the amount of omega-3 PUFAs, such as EPA and DHA [49]. In an earlier study from 2006, DHA and EPA levels were higher, at 18.2% and 8.2%, respectively. Additionally, the oleic acid content was lower at 15.6% [50]. This comparison thus shows that the evolution of farmed salmon diets impacts the levels of beneficial omega-3 PUFAs.

### 2.5. DLS Analysis of Liposome-Chondroitin Sulfate

The results of the characterization of L-CS are presented in Table 4. Surprisingly, it is the fraction with the least pure and heaviest CS (L-CSh1) that produces the smallest liposomes (65 nm). It would have been more logical to observe that the smallest would be the empty liposomes, but this is not the case (87 nm). Moreover, it is the smallest fraction with the lowest concentration of CS (L-CSl2) that shows the largest liposomes (94 nm). It is possible that the encapsulation or organization of compounds around the liposome compressed the liposome, making it smaller, just like in the work of Hanachi et al. where the encapsulation of peptides reduced the size of the liposome [51]. This could explain why the largest particles result in smaller liposomes. As for the ζ potential, the values are negative and centered around −20 mV. This negativity is favorable for particle stability due to the repulsion of negative charges. Zhang et al.’s work [52] shows L-CS with a size around 140 nm, which is larger than the values obtained here. This difference can be explained by the sonicator treatment, which creates smaller liposomes.

However, this smaller size is advantageous for the stability of the liposome and the release of its content at the level of cell membranes [53]. The same measurements were taken 7 days after formulation to study the evolution of size and ζ potential.

The sizes significantly increased. Only the value for the empty liposome showed little increase; this indicates that CS reduces the stability of the liposomes. The ζ potential also decreased significantly and nearly doubled for all the samples. This strong increase in negativity can be explained by the release of CS, which are negatively charged. The Polydispersity Index (PdI) also increased substantially due to the creation of new liposome sizes (following system destabilization) and the release of CS tending toward a multimodal size liposome distribution. Nonetheless, these values after 7 days are close to those found in the literature, around 140 nm [52].

### 2.6. Anti-Inflammatory Study of Chondroitin Sulfate-Liposomes on Human Chondrocytes

**Figure 3:**
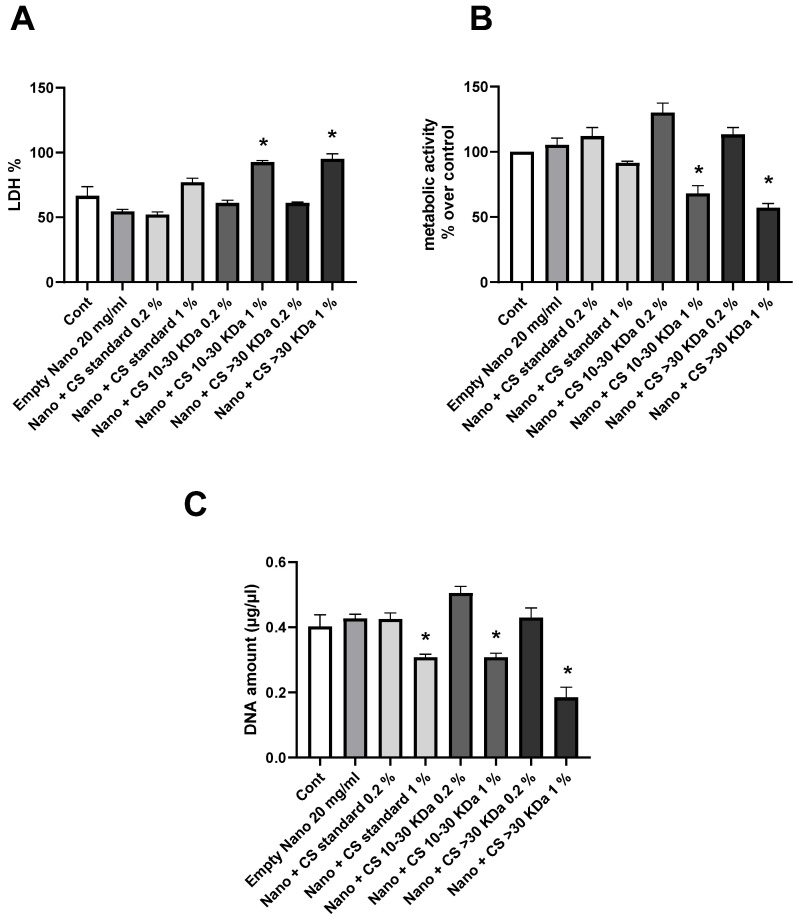
Biocompatibility of liposomes with CS with human chondrocytes. Human chondrocytes exposed to nanoliposomes (250 μg/mL) or nanoliposomes and CS extracts (250 μg/mL + 125 or 25 μg/mL) for 1 days. (**A**) Lactate Deshydrogenase (LDH) release determined as described under Section 3. Metabolic activity assessed using MTT assay. (**B**) Cell metabolic activity results on different membranes presented in % vs. control results (as 100%). (**C**) DNA concentrations measured to estimate proliferation of cells. Results shown are mean ± SD of at least four individual experiments. *: *p* < 0.01, compared to control.

The results presented in Figure 3 show the study of the biocompatibility of L-CS with chondrocytes. Cytotoxicity (A) was analyzed by Lactate Deshydrogenase (LDH) assay. The results indicate that an excessively high concentration of L-CS (1%) causes cell damage compared to the control. This effect is observed for all three categories of CS. At a concentration of 0.2%, no significantly higher toxicity levels compared to the control are noted. Cellular respiration was studied by evaluating metabolic activity with MTT (3-(4,5-dimethylthiazol-2-yl)-2,5-diphenyltetrazolium bromide) assay (B). Again, L-CS at 1% shows significantly lower mitochondrial respiration activity than the control. In contrast, L-CS at 0.2% results in a slight increase in mitochondrial activity compared to L-CS at 1%. Finally, the DNA quantity was analyzed to assess cell growth (C). L-CS at 1% leads to a significant decrease in DNA quantity, whereas L-CS at 0.2% shows a slight increase in DNA quantity compared to the control. These results demonstrate that using L-CS at 1% has a negative effect on cell growth and survival, while a lower concentration of 0.2% does not show this negative effect compared to the control and even shows a positive effect in some cases. Therefore, subsequent studies will be conducted using the 0.2% fraction of L-CS.

**Figure 4:**
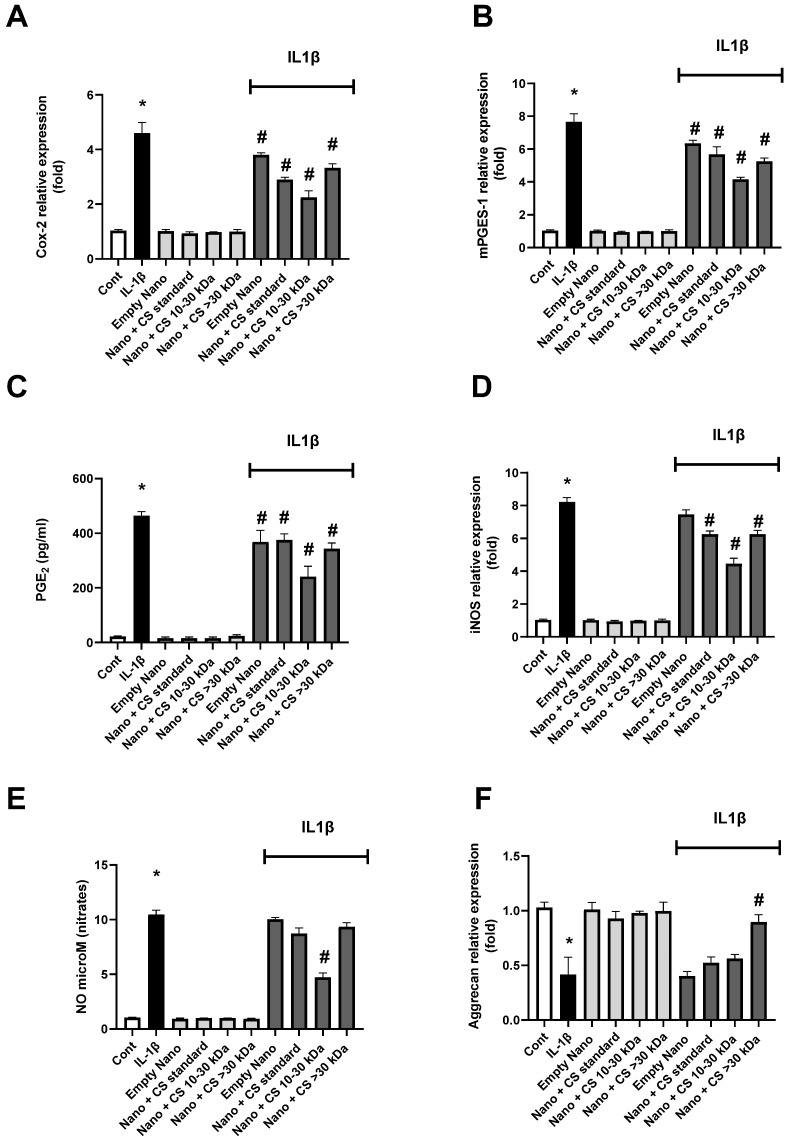
Effect of nanoliposomes/CS exposure on IL-1β-stimulated Cox/mPGES pathways. Human chondrocytes stimulated or not with 1 ng/mL IL-1β and exposed to nanoliposomes (250 µg/mL) or nanoliposomes and CS extracts (250 µg/mL + 25 µg/mL). For (**A**,**B**,**D**,**F**), culture conditions performed for 6 h for early inflammation markers (COX-2 and iNOS mRNA) and 24 h (Aggrecan and mPGES-1 mRNA). Total RNA extracted, then reverse transcribed into cDNA and analyzed by Real-Time Polymerase Chain Reaction (RT-PCR). Relative abundance of Cox-2, mPGES, iNOS, and Aggrecan mRNAs normalized to Retinitis Pigmentosa 29 (RP29) mRNA. Comparison made by using ΔCt method with fold value of reference = 1. Results shown are mean ± SD of at least four individual experiments (*: *p* < 0.01 vs. control; #: *p* < 0.01 versus IL-1β). For (**C**,**E**), culture conditions performed for 48 h for PGE2 and of nitrites (*: *p* < 0.01 vs. control; #: *p* < 0.01 vs. IL-1β).

The results of the effects of L-CS treatment on chondrocytes cultured under inflammatory conditions induced by IL-1β for different inflammatory pathways are presented in Figure 4. The expression of the COX-2 enzyme gene is shown in Figure 4A. The results indicate that each L-CS fraction significantly reduces inflammation compared to the control, with the reduction being most pronounced for the 10–30 kDa CS fraction. The expression of the mPGES-1 enzyme gene is analyzed in Figure 4B, showing results similar to those in Figure 4A. All L-CS fractions significantly reduce inflammation compared to the control, with an increased effect for the 10–30 kDa CS fraction. In Figure 4C, PGE2 concentration is measured, confirming the results of Figure 4A,B, with the 10–30 kDa CS fraction again showing the most substantial results. Figure 4D analyzes the expression of the iNOS gene, confirming the previous observations, as all L-CS fractions have a significant positive effect on IL-1β-induced inflammation. These results further emphasize that the 10–30 kDa CS fraction strongly reduces inflammation compared to other fractions.

Following the study of iNOS gene expression, nitrates produced by this enzyme during inflammation are measured in Figure 4E. These results are consistent with iNOS gene expression, as expected. Finally, Figure 4F tracks the expression of the aggrecan gene. In this case, inflammation reduces gene expression, and only the >30 kDa CS fraction at 0.2% shows a significant effect on increasing aggrecan expression compared to the controls. Interestingly, throughout the results, nanoliposomes without CS exhibit a significant intrinsic decrease in inflammatory markers, which is further enhanced in the presence of 0.2% CS, particularly for the 10–30 kDa fraction. This suggests a possible synergistic effect between the polar lipids in the liposomes and the CS in reducing inflammation.

These findings support those published by Chang et al. and Nguyen et al., demonstrating the anti-inflammatory effects of CS [54]. Here, we show that CS amplifies the intrinsic effects of empty nanoliposomes. Further studies are needed to confirm that these nanoliposomes extend the lifespan of CS and prevent their degradation in vivo in the joint site [55].

We also show that CS > 30 kDa have the strongest anti-inflammatory properties, which supports a recent study suggesting that low kDa CS (A and B) are present at non-inflamed sites, while CS < 30 kDa (C, D, and E) are more prevalent at highly inflamed sites [56].

**Figure 5:**
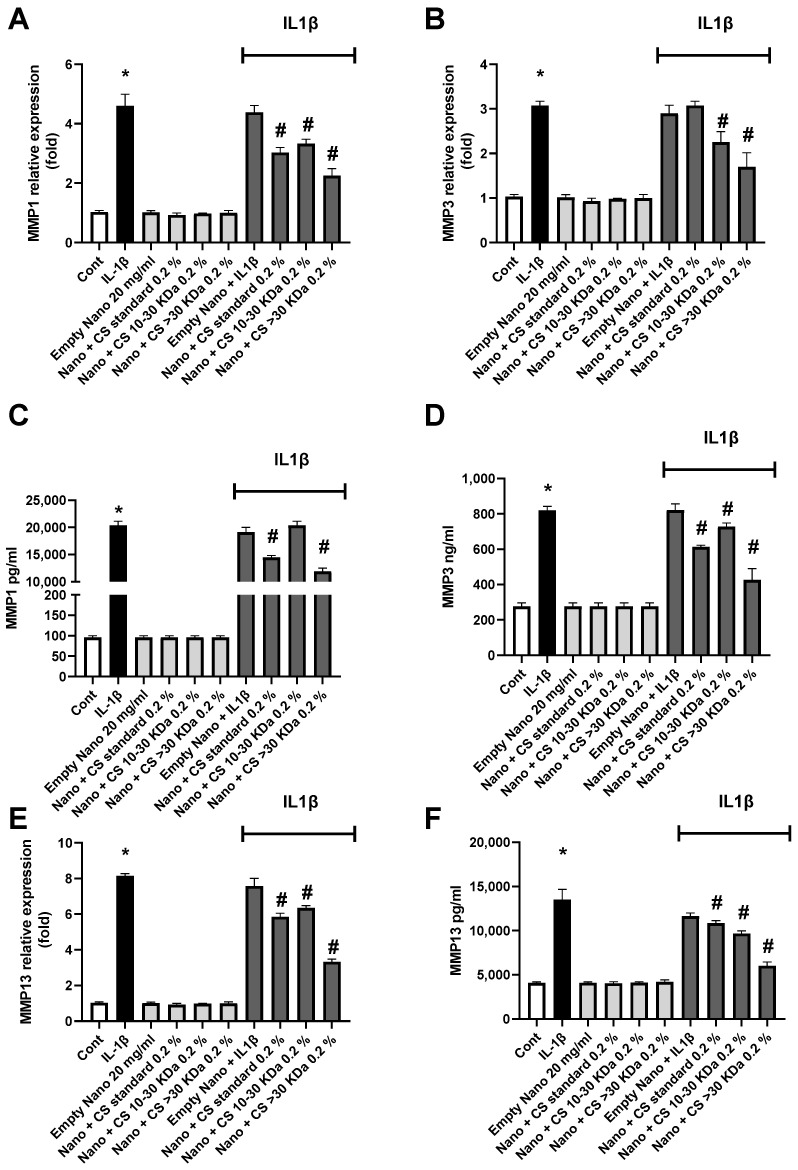
The effect of nanoliposomes/CS exposure on IL-1β-stimulated MMPs. Human chondrocytes were stimulated or not with 1 ng/mL IL-1β and exposed to nanoliposomes (250 µg/mL) or nanoliposomes and CS extracts (250 µg/mL+ 25 µg/mL). For (**A**,**B**,**E**), total RNA was extracted after 24 h stimulation then reverse transcribed into cDNA and analyzed by RT-PCR. The relative abundance of MMP1, MMP3, and MMP13 mRNAs was normalized to RP29 mRNA. Comparison was performed using the ΔCt method with the fold value of reference = 1. The results shown are the mean ± SD of at least four individual experiments (*: *p* < 0.01 vs. control; #: *p* < 0.01 vs. IL-1β). For (**C**,**D**,**F**) levels of MMP1, 3, and 13 after 48 h of stimulation (*: *p* < 0.01 vs. control; #: *p* < 0.01 vs. IL-1β).

To assess the anti-catabolic effect induced by IL-1β, the expression and quantity of metalloproteinases (MMPs) 1, 3, and 13—responsible for the turnover and/or degradation of the cartilage extracellular matrix—were evaluated. The expression and quantity of MMP1 are shown in Figure 5A,C, where both extracted L-CS fractions exhibit significant effects. Unlike previous studies on inflammatory pathways, it is the CS > 30 kDa 0.2% fraction that shows the best effect, although expression and production levels remain distant from those of the non-inflamed control. Another MMP, MMP3 was studied similarly in Figure 5B,D. Again, it is worth noting that the fraction containing the longest CS chains is much more effective on enzymatic activity than the 10–30 kDa fraction. This level is comparable to that of the control, indicating that this fraction is very effective on MMP3. Finally, the results of the same experiment on MMP13 are shown in Figure 5E,F. Similarly to the previous results, L-CS reduces both gene expression and MMP13 concentration. This efficacy is further enhanced with the CS > 30 kDa 0.2% fraction. Notably, in this case, the nanoliposomes without CS had no effect on these inflammation parameters, suggesting that polar lipids have little to no impact on MMP production in the cell renewal process.

The data presented in Figure 5 confirm the strong efficacy of the CS > 30 kDa 0.2% fraction in reducing MMP expression and concentration. Interestingly, the anti-catabolic effects of CS < 30 kDa appear to be stronger, contrary to what was observed for the anti-inflammatory side. This aspect will be explored in greater depth in future research. However, our results align with those obtained by Li et al.’s team [57].

## 3. Materials and Methods

### 3.1. Reagent

Fresh salmon (*Salmo salar*) heads were sourced from a local processing plant and stored at −20 °C. Before use, the heads were thawed overnight at 4 °C. Alcalase^®^ 2.4 L (EC.3.4.21.14a; Novozymes A/S, Bagsvaerd, Denmark), along with sodium hydroxide (NaOH) and hydrochloric acid (HCl), Neutrase^®^ 0.8 L (neutral protease, EC.3.4.24.4f, Novo Nordisk, Bagsvaerd, Denmark, food-grade enzyme), ethanol (C_2_H_6_O), chloroform (CHCl_3_), methanol, (CH_3_OH), hexane (C_6_H_14_), boron trifluoride (BF_3_), Tris base, CS marine standard, chondroitinase ABC, ammonium acetate, and acetonitrile were purchased from Sigma-Aldrich^®^ (Merck KGaA, Darmstadt, Germany) unless specified otherwise.

### 3.2. Extraction of Chondroitin Sulfate and Lipids from Salmon Heads (Salmo salar)

#### 3.2.1. Enzymatic Extraction

Total lipids and CS were extracted by green solvent-free processes from 12 kg heads of Atlantic salmon (*Salmo salar*), according to Linder et al. [58] with modifications. To extract the compounds of interest, the salmon heads will be subjected to a double enzymatic hydrolysis in order to extract the lipid fraction on one hand and the cartilaginous fraction containing chondroitin sulfate present in the salmon head on the other hand.

The substrate was thawed before being placed in a thermostatic reactor under agitation with water 1:1 (*v*/*v*). After addition of the enzyme Neutrase^®^ 0.8 L (neutral protease, EC.3.4.24.4f, Novo Nordisk, Bagsvaerd, Denmark, food-grade enzyme) to the reaction medium (50 °C, pH 7.5), the reaction is controlled by the “pH-stat” method for 15 min. At the end of hydrolysis, the enzyme is inactivated (90 °C, 10 min). This first step allows the cartilaginous fraction of the head to be recovered after filtration, free from the lipid and protein fractions.

The CS-rich cartilaginous parts of the salmon nasal fraction are then subjected to a second hydrolysis to release the CS in the presence of Alcalase^®^ 2.4 L protease (Novo Nordisk, Bagsvaerd, Denmark) for 40 min at 55 °C and pH 8. The reaction is controlled by the pH-stat method, as with the first hydrolysis, by the addition of a sodium hydroxide solution (2N). The enzyme is then inactivated by heat treatment at 90 °C for 10 min.

Both reaction mixtures are filtered with an inox filter of 300 mesh to remove the solid structures and centrifuged using a Beckman Coulter^®^ Rotor J-10 (Beckman Coulter^®^, Brea, CA, USA) at 20 °C, 14,334× *g* for 20 min to obtain four different phases: oil, emulsified lipids, proteolysis, and a heavy fraction rich in peptides. The peptide phase is freeze-dried with a Christ^®^ Beta 1–8 Lyophilisator (Grosseron, France) and stored at −20 °C under vacuum in plastic bags.

#### 3.2.2. Purification of CS Fraction

Proteolytics CS are precipitated to ethanol using the method presented in the review of José Antonio Vasquez et al. [2]. A solution of 50% ethanol diluted in water with 0.2 M NaOH is added to the freeze-dried peptidic fraction in proportions of 1:1.

The mixture is stirred for 1 h at 20 °C, before being centrifuged at 6371× *g* with J-10 rotor for 20 min. The unit containing the precipitated CS is recovered and re-suspended in distilled water. The precipitation step is repeated 3 times before the samples are freeze-dried. An AMICON^®^ (Amicon^®^ stirred cell, Merck KGaA, Darmstadt, Germany) with 200 mL membrane filtration stage with Ultracel^®^ cellulose membranes (Ultrafiltration disks, Merck, Jaffrey, NH, USA) of 10 and 30 kDa yields were used. Then, the 10 to 30 kDa and greater than 30 are freeze-dried. These fractions are diluted in distilled water at a concentration of 30 g/L to prevent clogging of the membrane. The separate phases are recovered and re-suspended in water before being freeze-dried and stored at 4 °C before analysis.

#### 3.2.3. Separation of Lipid Classes and Purification of Phospholipids

The different lipid fractions, neutral and polar, were separated by centrifugation (20 °C, 20 min, 14,334× *g*) and purified using the appropriate solvents. Regarding polar lipids, a freeze-drying step is performed to remove water given the amphipathic character of the polar lipids.

The recovery of the residues contained in the peptide fraction obtained as a result of hydrolysis is carried out according to Folch et al. [59]. The powder resulting from the hydrolysis of the heads is solubilized in a mixture of chloroform/methanol (99.9%) in proportions 2:1 (*v*/*v*), respectively. The solution is then filtered through a sintered glass (Robu-Glas^®^ borosilicat por. 4, Hattert, Germany) under vacuum. The lipid solution is then evaporated at 40 °C in the dark and under nitrogen by Rotavapor R-205, with a vacuum connector V-800, and a pumpV-500 BUCHI (BUCHI Sarl, Villebon-sur-Yvette, France). The recovered lipid fractions are subjected to glacial acetone precipitation at −20 °C to separate the neutral and polar lipid fractions according to Zhu et al. [60].

### 3.3. FT-IR Spectroscopy Analysis of CS and Lipids

The analyses are performed in Infrared to Fourier Transform Attenuated Total Reflection (FTIR-ATR) mode at 22 °C in a Tensor 27 mid-FTIR Bruker spectrometer (Brüker, Karlsruhe, Germany), equipped with a DTGS detector (Mercury-Cadmium-Tellure, Brüker, Karlsruhe, Germany) and an ATR A537-L11 optical cell. The diaphragm was set to 6 mm, with a scanning speed of 10 KHz. There were 128 scans performed by analysis, from 400 to 4000 cm^−1^ with a spectral resolution of 6 cm^−1^ according to Chihaoui et al. [61] with modifications.

### 3.4. Digestion of CS and Semi-Quantitative Analysis of Δdi-4S and Δdi-6S by HPLC-MS

CS disaccharides were obtained by chondroitinase ABC digestion according to Takeda et al. [62]. Then, 1 mg of the extracted CS samples was diluted in 1 mL of Tris-HCl buffer at pH 8. After the addition of chondroitinase ABC, the mixture is placed at 37 °C for 24 h. The sample is centrifugated, and supernatant is collected for analysis. The same procedure is realized with a marine CS commercial standard, which is becoming a disaccharide reference mixture for the calibration curve.

The qualitative and semi-quantitative analysis of Δdi-4S and Δdi-6S were conducted on an HPLC-MS system (Thermo Scientific, San Jose, CA, USA) consisting of a quaternary solvent delivery pump connected to a photodiode array detector (PDA) and a LTQXL mass spectrometer equipped with an atmospheric pressure ionization interface operating in electrospray negative ion mode (ESI–).

Ten microliters of chondroitin hydrolysis sample were injected on a Hypercarb column (100 × 2.1 mm, 5 μm, Thermo Fisher Scientific) maintained at 40 °C during the run. Mobile phase A was ammonium acetate in water at pH 11 and mobile phase B was pure acetonitrile. The flow rate was set at 0.2 mL/min. Sulfate disaccharides were eluted by an isocratic step at 6% B for 1.5 min, followed by a linear gradient from 6 to 11% B over 9 min.

Mass spectrometry (MS) conditions were as follows: spray voltage was set at −4.5 kV; source gasses were set (in arbitrary units. min^−1^) for sheath gas, auxiliary gas and sweep gas at 29, 0.5, and 1, respectively; capillary temperature was set at 280 °C; capillary voltage was set at −45 V; and tube lens, split lens, and front lens voltages were set at −77 V, 60 V, and 9 V, respectively. Ion optics parameters were optimized by automatic tuning using ΔCS-4S and ΔCS-6S present in chondroitin hydrolysis solution (0.5 g/L) infused in mobile phase at a flow rate of 5 μL/min. Full scan MS (100 to 1000 *m*/*z*) and MS/MS spectra were performed on an LTQ analyzer (Linear Trap Quadrupole). Raw data were processed using the XCALIBUR software program (version 2.1, http://www.thermoscientific.com (accessed on 15 October 2024).

### 3.5. TLC-FID (Iatroscan^®^) Analysis of Lipids

The lipid fraction analysis was performed by Iatroscan^®^ MK-5 thin-layer chromatography–flame ionization detection (TLC-FID) (Iatron Laboratories Inc., Tokyo, Japan). The samples are diluted in a mixture of chloroform/methanol (99.9%; 2:1 *v*/*v*) to obtain a concentration of 5 mg/mL. A sample (1 µL) was deposited in tripliquat on chromarods S-III (Iatroscan laboratory Inc, Tokyo, Japan). The migration was conducted for 20 min in a solution of hexane/diethyl ether/formic acid (80:20:0.2 *v*/*v*/*v*), then oven-dried for 1 min at 100 °C, and finally scanned by the Iatroscan^®^ analyzer. A second migration in a chloroform/methanol/ammonia mixture (65/35/5 *v*/*v*/*v*) allows for the detection of the different phospholipids. Hydrogen and air flows are maintained at 160 and 2 mL per minute at 20 °C, respectively. Peak recording and integration were performed with ChromStar^®^ software 4.14 (SCPA, Weyhe-Leeste, Germany). Triglyceride, diglyceride, and monoglyceride standards were used to perform the calibration range (Sigmal-Aldrich, St. Louis, MO, USA).

### 3.6. Fatty Acid Composition Analysis by GC-FID

The fatty acid composition of the different lipid classes is achieved by gas chromatography coupled with a flame ionization detector (GC-FID) according to the method of Li et al. [33] with modifications. The samples are methylated according to the technique of Morrisson and Smith [63]. Esterification is performed by diluting 0.1 g of lipids in 1.5 mL of hexane and 1.5 mL in a mixture of boron/methanol trifluore (8% of BF_3_). The samples were analyzed by CPG-FID SHIMADZU 2010 (Kyoto, Japan), coupled with a flame ionization detector and equipped with a silica capillary column (60 m, 0.2 mm i.d. 0.25 µm film thicknesses, SPTM2380 Supelco, Bellfonte, PA, USA). The injector and detector temperature are set at 250 °C. During the first 3 min, the column temperature reaches 120 °C. It is then increased to 180 °C at a speed of 40 °C per minute for 2 min, then maintained at 220 °C for 25 min. The data were performed with Shimadzu “GC solution” software 2.41.00 (Kyoto, Japan). The identification of fatty acids is carried out using standard mixtures from marine PUFA1 and animal PUFA2 sources, respectively (Supelco, Sigma–Aldrich, Bellefonte, PA, USA).

### 3.7. Formulation of L-CS

Liposomes are formulated according to the thin lipid film hydration method according to Bingham in 1961. For the lecithin concentration of liposomal dispersion to be 2%, 160 mg of lecithin is accurately weighed and diluted in chloroform/methanol (2:1 *v*/*v*) in a flask according to Hanachi et al. [51]. The thin film is formed by evaporation with Rotavapor R-205, with a vacuum connector V-800, and a pumpV-500 BUCHI (BUCHI Sarl, Villebon-sur-Yvette, France). The film is then rehydrated overnight at 4 °C under agitation with a solution containing CS diluted in 8 mL of ultrapure water. The samples are then sonicated with a sonicator (sonicator probe, Sonics & Materials Inc., CT, USA) for 8 min (1 s «on»/1 s «off») at 40 °C, 40% amplitude, and at a frequency of 20 kHz.

Different types of liposomes are formed for characterization and cell study: empty liposomes (L-E); liposomes containing the marine CS standard with two concentrations: 1.0% (L-CSs1) and 0.2% (L-CSs2); and liposomes containing CS extracted from salmon heads greater than 30 kDa at 1% (L-CSh1) and 0.2% (L-CSh2) and between 10 and 30 kDa at 1.0% (L-CSl1) and 0.2% (L-CSl2).

### 3.8. DLS Analysis of L-CS

To analyze the size, Polydispersity Index (PdI), and liposome charge (potential ζ), the sample was analyzed by DLS (Zetasizer Nano ZS, Malvern, UK). For this, the liposome sample is diluted twice in ultra-pure water. The dilution is transferred to a micro vessel (UV-Cuvette micro, BRAND GMBH + CO. KG, Wertheim, German) for size measurement using Zetasizer software V2.41 (ZetaSizer; Malvern Instrument Ltd., Malvern, UK). For load measurement, the sample is analyzed in a capillary vessel DTS1070 (Malvern Instrument Ltd.) using the Zetasizer software. Size, potential ζ, and PdI were measured at 25 °C, with an absorbance of 0.01 at a fixed diffusion angle of 173° and a refractive index of 1.471.

### 3.9. Anti-Inflammatory Study of CS-Liposomes on Human Chondrocytes

#### 3.9.1. Collection and Culture of Chondrocytes

All specimen collection and all procedures conducted were approved by the Ethics Committee of the Nancy University Hospital (CHU) (agreement #UF 9607-CPRC 2005) and conducted in conformity with the declaration of Helsinki. Femoral condyles and tibial plateaus were obtained from 6 osteoarthritis patients (aged 70 ± 9 years, mean ± SD; M/F: 3/11). For in vitro studies, chondrocytes were released from cartilage pieces by sequential enzymatic digestions with collagenase (1.5 mg/mL) and pronase^®^ (2 mg/mL). They were cultured in DMEM (Dulbecco’s Modified Eagle Medium, Gibco^TM^, Life Technologies GmbH, Darmstadt, Germany) containing 4.5 g/L glucose and 10% fetal calf serum (Gibco, Villebon-sur-Yvette, France) for the first 24 h, and then in DMEM containing 1 g/L glucose and 10% fetal calf serum until 70% confluence and then stimulated. The cells were collected after 6 and 24 h for RNA extraction, and supernatants after 48 h for ELISAs.

#### 3.9.2. Study Design

Human chondrocytes were stimulated with 1 ng/mL of IL-1β for 6, 24, or 48 h with or without 250 μg/mL of liposomes empty or with CS encapsulated at 125 μg/mL (1%) or 25 μg/mL (0.2%) in the culture medium. Supernatants were frozen immediately and kept at −80 °C until use and were assayed individually for the nitrites assay and specific ELISAs. As the transcription of the inflammation markers COX-2 and iNOS was early [55], we first measured their expression after 6 h of cell culture. Then, we measured the expression of mPGES-1 [64] after 24 h. Finally, as PGE2 is an end product of the COX-2 and mPGES-1 pathways, and NO for iNOS [65,66], they were quantified after 48 h of cell culture.

#### 3.9.3. Biocompatibility Assays

To evaluate the impact of nanoliposomes on cell behavior, different parameters were estimated: cytotoxicity, cell metabolic activity, and cell proliferation.

The cytotoxicity test was performed using the Cytotoxicity Detection KitPLUS (LDH) (#04744926001; Roche, Boulogne-Billancourt, France) according to the manufacturer’s instructions. This assay is based on the measurement of LDH activity released from the cytosol of damaged cells. Three controls are included: background control (assay medium), low control (untreated cells), and high control (maximum LDH release). The absorbance was read on a spectrophotometer at 490 nm (Varioskan^®^ Flash, Thermo Scientific, Illkirch, France). To determine the experimental absorbance values, the average absorbance values of the triplicate samples and controls were calculated and subtracted from the absorbance values of the background control. The percentage of cytotoxicity was determined over the value of the high control (fixed to 100).

Cell proliferation was assessed after 3, 5, or 7 days of chondrocyte culture using Hoechst assay, which allows for cell DNA quantification. Briefly, chondrocytes were harvested from 12-well plates and suspended in 100 µL of Hoechst buffer (10 mM TRIS, 1 mM EthyleneDiamineTetraacetate Acid (EDTA), and 0.1 M of NaCl, pH 7.4) before 5 series of freezing (liquid nitrogen)/thawing (60 °C, 5 min) cycles for lysing cells and releasing their DNA into solution. Black fat-bottom plates with a low fluorescent background were used to perform the assay and a calf thymus DNA standard curve was used for quantification. The samples were mixed with 2 µL of the Hoechst solution (0.1 µg/mL in final concentration) and the measurements of the DNA samples and standards were performed by fluorescence spectrophotometry (360 nm excitation/460 nm emissions, Varioskan^®^ Flash, Thermo, Illkirch, France). The DNA concentration (µg/mL) of each sample was based on its fluorescence measurement relative to the standard curve.

Cell metabolic activity was measured using MTT (3-(4,5-dimethylthiazol-2-yl)-2,5-diphenyltetrazolium bromide) assay as described elsewhere. Then, 50 µL of the MTT solution was added to 200 µL of the cell culture medium. Briefly, chondrocytes were incubated for 4 h (5% CO_2_, 95% humidity at 37 °C) to allow the yellow dye to be transformed into blue formazan crystals by the mitochondrial dehydrogenases. The supernatant was removed, and this insoluble product was protected from light and dissolved by the addition of 200 µL Dimethylsulfoxid (DMSO) and gently mixed at 37 °C for 5 min. The supernatants were removed, protected from light, centrifuged, and their absorbance was read within 30 min using a Varioskan^®^ Flash (Thermo Fisher Scientific, Illkirch, France) at 540 nm. The control condition for chondrocyte metabolic activity was used as the reference value.

#### 3.9.4. Nitrites Assay

NO production was estimated spectrophotometrically by measuring the accumulation of nitrites in culture supernatants by the Griess reaction. Briefly, 100 µL of culture supernatant were mixed with 100 µL of the Griess reagent (1% of sulfanilamide in 2.5% H_3_PO_4_ and 0.1% of N-Naphtylethylenediamine dihydrochloride in H_2_O, *v*/*v*) for 5 min at room temperature in microtiter plates. The absorbance was measured at 550 nm with a microplate reader (Multiskan, Labsystems, Cergy Pontoise, France), and nitrite concentrations were calculated with a standard curve of sodium nitrite ranging from 0 to 50 µM. The limit of quantification of this method was determined to be 1 µM of nitrites.

#### 3.9.5. Immuno-Enzymatic Assays (ELISAs)

As mentioned above, the effect of IL-1β and/or nanoliposomes on the level of several proteins secreted by chondrocytes was controlled by enzyme-linked immunosorbent assays (ELISAs). After stimulations, supernatants were collected and centrifuged 5 min at 600 g and analyses were performed according to the kit manufacturer’s instructions for MMP1, MMP3, and MMP13 levels (DuoSet^®^ ELISA, R&D systems, Abingdon, UK—Human Total MMP1, Human Total MMP3, and Human Pro-MMP13) and PGE2 levels (Prostaglandin E2 Parameter Assay Kit, Bio-Techne, Abingdon, UK).

#### 3.9.6. RNA Extraction and Reverse Transcription-Polymerase Chain Reaction Analysis

Total RNA was isolated using RNeasy plus mini kit^®^ (Qiagen, Hilden, Germany), which allows for the total removal of genomic DNA with an on-column DNase. Then, 500 ng of the total RNA were reverse-transcribed for 90 min, at 37 °C in a 20 µL reaction mixture containing 5 mM dNTP, 0.2 µg/µL random hexamer primers, 250 mM Tris-HCl-pH 8.3, KCl 375 mM, MgCl_2_ 15 mM, and 200 units of Moloney Murine Leukemia Virus reverse transcriptase (Invitrogen, Austin, TX, USA).

#### 3.9.7. Real-Time Quantitative Polymerase Chain Reaction

Real-time PCR was performed by the Step One Plus (Applied Biosystems, France) technology using specific primers (Table 5) and iTAQ SYBRgreen master mix system (Biorad, Steenvoorde, Combes-La-Ville, France). All reagents used for RT-PCR were added at the concentrations recommended by the manufacturer. Melting curve was performed to determine the melting temperature of the specific PCR products and, after amplification, the product size was checked on a 1% agarose gel stained with Gel Red (Biotium, Interchim, Montluçon, France). The mRNA levels of the gene of interest and of the ribosomal protein 29 (RPS29), chosen as a housekeeping gene in order to normalize gene expression, were determined in parallel for each sample. Quantification was determined using the ΔΔCt method and the results were expressed as fold expression over the control.

#### 3.9.8. Statistical Analysis

All experiments were performed and analyzed in triplicates. The results are expressed as the mean ± SEM. Statistical analyses were performed with GraphPad Prism 6 (GraphPad Software Inc., San Diego, CA, USA) using either one-way ANOVA followed by Bonferroni’s post hoc test or unpaired T-test, with Welch’s correction when variances were significantly different. *p* values lower than 0.05 were considered significant for physicochemical analyses and 0.01 for biomolecular analyses.

**Figure 6:**
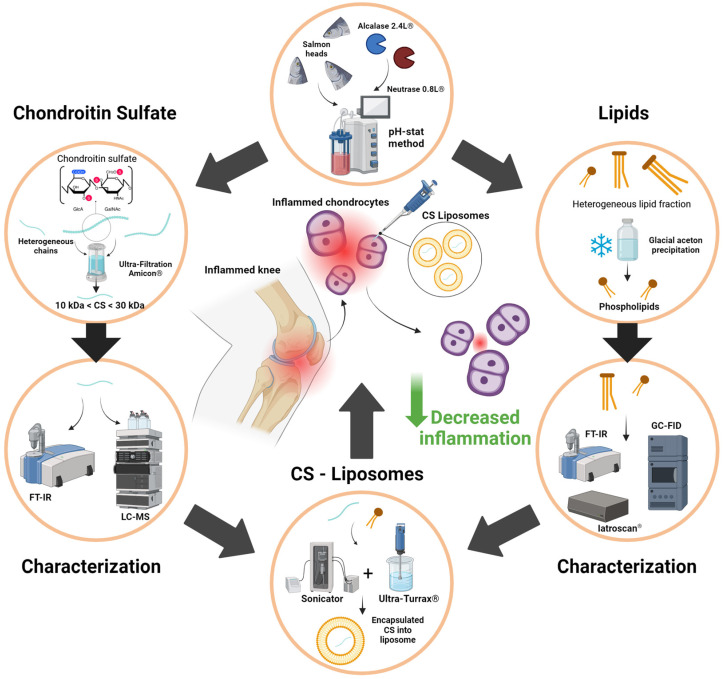
Recapitulative graphical abstract of study design and objectives.

Figure 6 summarizes the valorization of by-products from the fishing industry, particularly the use of salmon heads (*Salmo salar*) as a source of phospholipids naturally rich in LC-PUFAs and chondroitin sulfate present in the nasal region of cartilage tissue. The results showed that CS, in synergy with the LC-PUFAs present in the phospholipids of the liposomes, played a positive role in combating human chondrocyte inflammation even at low concentrations.

## 4. Conclusions

As a result of these findings, it is possible to formulate liposomes loaded with chondroitin sulfate, capable of reducing inflammation in joint cells, using a single co-product from salmon (*Salmo salar*). The various characterization techniques used confirmed that chondroitin sulfate and polar lipids were successfully extracted, separated, and concentrated. These biomolecules were used to form liposomes, which, through a synergistic effect between the polar lipids and the chondroitin sulfate, were able to reduce the expression of inflammation markers in human chondrocytes. Moreover, the CS fraction showed different actions on inflammation with a good effect of 10–30 kDa for reducing pathway inflammation markers and the fraction > 30 kDa for the stimulation of aggrecan gene expression and reducing gene expression and the concentration of MMP enzymes. The formulation of such products fully meets contemporary demands with a dual benefit: offering a solution to growing conditions such as osteoarthritis and using food by-products as part of an anti-waste approach. The next steps following this study would be to more precisely characterize the extracted chondroitin sulfate in terms of length and composition. It could also be relevant to scale up production, increase the extraction yields of the different molecules through experimental designs, or test this anti-inflammatory effect on other types of cells.

## Figures and Tables

**Table 2:** Wavenumber (cm^−1^) of FT-IR spectra of CS and lipids extracted from *Salmo salar* heads by enzymatic hydrolysis.

Chondroitin Sulfate		Lipids	
Bond	Wavenumber (cm^−1^)	Bond	Wavenumber (cm^−1^)
-OH, H_2_O	3400	-H-OO	3250
-CH	2920	-CH	3005
-CH	2850	-CH_3_	2958
-C-O	1625	-CH_2_	2920
-NH	1550	-CH_3_	2850
-CO	1373	-CH_2_, -CH_3_	1450
-CO	1410	Ester -CO	1080
-SO	1220	(-CH_2_–, -HC=CH-(*cis*))	720

**Table 3:** Fatty acids composition of different lipid fractions extracted (% of total fatty acids) from *Salmo salar* heads by enzymatic hydrolysis.

Fatty Acids	TAG	Glycolipids	Phospholipids
C14:0	3.68 ± 0.03	1.68 ± 0.10	2.71 ± 0.14
C15:0	0.12 ± 0.01	0.27 ± 0.01	0.25 ± 0.25
Iso C16:0	0.22 ± 0.01	0.49 ± 0.01	0.40 ± 0.01
C16:0	10.80 ± 0.01	18.97 ± 0.05	17.15 ± 0.30
C18:0	2.27 ± 0.04	5.53 ± 0.01	4.90 ± 0.08
C20:0	0.57 ± 0.02	1.88 ± 0.02	2.20 ± 0.01
**Σ Saturates**	**17.66**	**30.02**	**27.61**
C16:1 n-9	4.99 ± 0.01	2.96 ± 0.06	2.52 ± 0.07
C18:1 n-9	28.88 ± 0.12	21.02 ± 0.01	18.10 ± 0.19
C18:1 n-7	0.23 ± 0.01	0.33 ± 0.01	0.31 ± 0.01
C20:1 n-11	7.99 ± 0.13	4.19 ± 0.09	3.57 ± 0.15
C20:1 n-9	1.46 ± 0.07	0.57 ± 0.08	0.63 ± 0.11
C20:1 n-7	0.17 ± 0.01	0.19 ± 0.03	0.25 ± 0.05
C22:1 n-9	0.38 ± 0.02	0.40 ± 0.02	0.55 ± 0.04
**Σ Monoenes**	**44.10**	**29.66**	**25.93**
C16:2 n-6	0.19 ± 0.01	0.34 ± 0.05	0.32 ± 0.01
C16:2 n-4	0.36 ± 0.01	0.72 ± 0.01	0.54 ± 0.01
C18:2 n-7	0.30 ± 0.02	0.16 ± 0.01	0.14 ± 0.01
C18:2 n-6	7.24 ± 0.01	3.96 ± 0.01	3.66 ± 0.04
C18:2 n-4	0.18 ± 0.01	0.26 ± 0.02	0.20 ± 0.01
**Σ Dienenes**	**8.27**	**5.44**	**4.86**
C16:3 n-4	0.55 ± 0.02	0.53 ± 0.01	0.47 ± 0.02
C18:3 n-6	0.20 ± 0.02	0.12 ± 0.01	0.13 ± 0.01
C18:3 n-4	2.70 ± 0.10	1.63 ± 0.01	1.59 ± 0.02
C18:3 n-3	8.33 ± 0.04	4.63 ± 0.01	3.90 ± 0.05
C18:3 n-1	0.23 ± 0.01	0.25 ± 0.01	0.23 ± 0.01
**Σ Trienenes**	**12.01**	**7.16**	**6.32**
C16:4 n-1	0.27 ± 0.05	0.36 ± 0.02	0.31 ± 0.09
C18:4 n-3	0.59 ± 0.02	0.61 ± 0.01	0.59 ± 0.02
C22:4 n-6	0.69 ± 0.01	2.93 ± 0.01	2.56 ± 0.12
**Σ Tetraenes**	**1.55**	**3.90**	**3.46**
C20:5 n-3	4.89 ± 0.03	5.79 ± 0.02	7.44 ± 0.03
C21:5 n-3	0.44 ± 0.01	0.63 ± 0.01	0.84 ± 0.07
C22:5 n-3	1.82 ± 0.04	1.94 ± 0.05	2.31 ± 0.04
**Σ Pentaenes**	**7.15**	**8.36**	**10.59**
C22:6n-3	7.12 ± 0.03	13.64 ± 0.02	19.38 ± 0.40
Σ n-6	8.32	7.35	9.06
Σ n-3	23.19	27.24	34.46
n-6/n-3	0.36	0.27	0.26
EPA/DHA	0.69	0.42	0.38

**Table 4:** Size, ζ potential, and Polydispersity Index (PdI) of different liposome formulations with or without CS.

	Day 0			Day 7		
Samples	Size (nm)	ζ Potential (mV)	PdI	Size (nm)	ζ Potential (mV)	PdI
L-E	87.91 ± 3.19	−20.60 ± 0.79	0.26 ± 0.01	95.12 ± 9.44	−28.60 ± 0.66	0.268 ± 0.07
L-CSl1	82.07 ± 0.98	−20.30 ± 0.76	0.21 ± 0.01	123.56 ± 2.54	−39.00 ± 0.46	0.282 ± 0.01
L-CSl2	94.01 ± 0.99	−21.20 ± 1.20	0.21 ± 0.01	148.33 ± 2.80	−42.60 ± 0.58	0.247 ± 0.03
L-CSh1	65.76 ± 1.95	−16.20 ± 0.32	0.25 ± 0.01	74.41 ± 4.83	−36.10 ± 0.43	0.310 ± 0.06
L-CSh2	84.59 ± 1.12	−19.90 ± 0.75	0.22 ± 0.01	173.33 ± 2.41	−37.30 ± 0.28	0.364 ± 0.01
L-CSs1	74.76 ± 0.34	−19.30 ± 0.10	0.24 ± 0.01	144.13 ± 3.65	−47.70 ± 0.80	0.254 ± 0.01
L-CSs2	84.03 ± 0.88	−15.50 ± 0.43	0.22 ± 0.01	132.96 ± 2.55	−38.60 ± 0.55	0.389 ± 0.01

L-E: empty liposomes; L-CSh1: liposomes with more than 30 kDa CS at 1%; L-CSh2: liposomes with more than 30 kDa CS at 0.2%; L-CSl1: liposomes with CS between 10 and 30 kDa CS at 1%; L-CSl2: liposomes with CS between 10 and 30 kDa CS at 0.2%; L-CSs1: liposomes with standard CS at 1%; L-CSs2: liposomes with standard CS at 0.2%.

**Table 5:** Sequences of specific primers for RT-PCR analyses.

Genes	Sequences 5′-3′
Agg	Fwd: CTC-TAA-CCG-CCA-CGG-TCT-GA
	Rev: ACT-AGC-ATG-ATT-GGT-ATC-AC
COX-2	Fwd: GCT-GGA-ACA-TGG-AAT-TAC-CCA
	Rev: CTT-TCT-GTA-CTG-CGG-GTG-GAA
mPGES-1	Fwd: TGG-TCA-TCA-AGA-TGT-ACG-TGG-T
	Rev: GGG-TCG-CTC-CTG-CAA-TAC-T
iNOS	Fwd: TGC-AAT-GAA-TGG-GGA-AAA-AG
	Rev: ATT-CTG-CTG-CTT-GCT-GAG-GT
MMP1	Fwd: AGG-TCT-CTG-AGG-GTC-AAG-CA
	Rev: CTG-GTT-GAA-AAG-CAT-GAG-CA
MMP3	Fwd: GCA-GTT-TGC-TCA-GCC-TAT-CC
	Rev: GAG-TGT-CGG-AGT-CCA-GCT-TC
MMP13	Fwd: TGG-TGG-TGA-TGA-AGA-TGA-TTT
	Rev: TCT-AAG-CCG-AAG-AAA-GAC-TGC
RP29	Fwd: GGG-TCA-CCA-GCA-GCT-GTA-CT
	Rev: CCG-ATA-TCC-TTC-GCG-TAC-TG

Agg—aggrecan; COX-2—cyclooxygenase-2; mPGES-1—microsomal prostaglandin E2 synthase-1; iNOS—inducible nitric oxide synthase; Fwd—forward primer; RT-PCR—real-time polymerase chain reaction; Rev—reverse primer; RP—ribosomal protein; MMP1, 3, and 13—metalloproteinase 1, 3, and 13.

**Figure A1:**
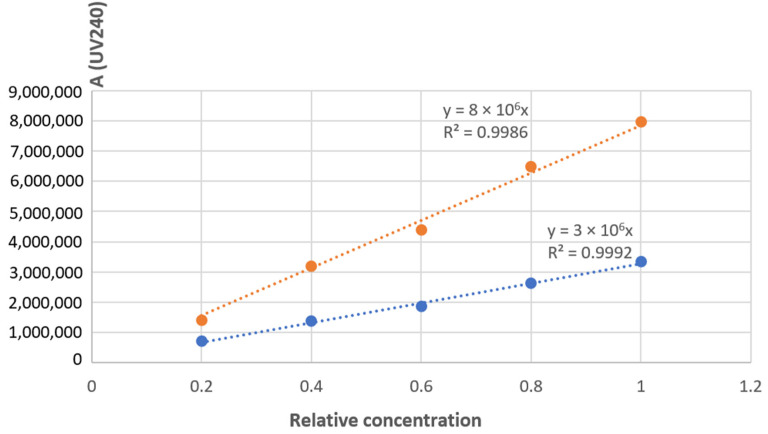
Calibration range of 4S and 6S isomers.

## Data Availability

The original contributions presented in the study are included in the article, further inquiries can be directed to the corresponding author.
